# Retraction Note: LncRNA CTBP1-AS2 sponges miR-216a to upregulate PTEN and suppress endometrial cancer cell invasion and migration

**DOI:** 10.1186/s13048-020-00754-0

**Published:** 2020-12-23

**Authors:** Qing-an-zi Wang, Yongxiu Yang, Xiaolei Liang

**Affiliations:** Department of Obstetrics and Gynecology, Key Laboratory for Gynecologic Oncology Gansu Province, the First Hospital of Lanzhou University, Lanzhou City, Gansu Province 730000 PR China

**Retraction Note: J Ovarian Res 13, 3 (2020)**

10.1186/s13048-020-00639-2

The Editors-in-Chief has retracted this article [1] due to lack of evidence that the study has received ethics approval. After publication it has come to the Editor’s attention that in the body of the article the authors state that their study was approved by the Ethics Committee at the First Hospital of Lanzhou University. However, in the Ethics declarations section, the authors state that their study was approved by the Ethics Committee of the Maternity and Child Care Center of Liuzhou. The authors have not provided documentation of approval from an ethics committee for this study. Yongxiu Yang and Qing-an-zi-Wang agree with this retraction. Xioalei Liang have not responded to any correspondence regarding this retraction.

## References

[1] Wang Q, Yang Y, Liang X (2020). LncRNA CTBP1-AS2 sponges miR-216a to upregulate PTEN and suppress endometrial cancer cell invasion and migration. J Ovarian Res.

